# Effects of a temporary suspension of community-based health insurance in Kwara State, North-Central, Nigeria

**DOI:** 10.11604/pamj.2022.41.10.27978

**Published:** 2022-01-05

**Authors:** Oladimeji Akeem Bolarinwa, Tanimola Makanjuola Akande, Wendy Janssens, Kwasi Boahene, Tobias Rinke de Wit

**Affiliations:** 1 Department of Epidemiology and Community Health, University of Ilorin, Ilorin, Nigeria,; 2 Amsterdam Institute for Global Health and Development, Vrije Universiteit, Amsterdam, Netherlands,; 3 PharmAccess Foundation, Paasheuvelweg, Amsterdam, Netherlands

**Keywords:** Community-based, health insurance, suspension, Kwara

## Abstract

**Introduction:**

a subsidized community health insurance programme in Kwara State, Nigeria was temporarily suspended in 2016 in anticipation of the roll-out of a state-wide health insurance scheme. This article reports the adverse consequences of the scheme´s suspension on enrollees´ healthcare utilization.

**Methods:**

a mixed-methods study was carried out in Kwara State, Nigeria, in 2018 using a semi-quantitative cross-sectional survey amongst 600 former Kwara community health insurance clients, and in-depth interviews with 24 clients and 29 participating public and private healthcare providers in the program. Both quantitative and qualitative data were analyzed and triangulated.

**Results:**

most of former enrollees (95.3%) kept utilizing programme facilities after the suspension, mainly because of the high quality of care. However, majority of the enrollees (95.8%) reverted to out-of-pocket payment while 67% reported constraints in payment for healthcare services after suspension of the program. In the absence of insurance, the most common coping mechanisms for healthcare payment were personal savings (63.3%), donations from friends and families (34.7%) and loans (11.8%). Being a male enrollee (odd ratio=1.61), living in a rural community (odd ratio =1.77), exclusive usage of Kwara Community Health Insurance Programme (KCHIP) prior to suspension (odd ratio=1.94) and suffering an acute illness (odd ratio=3.38) increased the odds of being financially constrained in accessing healthcare.

**Conclusion:**

after the suspension of the scheme, many enrollees and health facilities experienced financial constraints. These underscore the importance of sustainable health insurance schemes as a risk-pooling mechanism to sustain access to good quality health care and financial protection from catastrophic health expenditures.

## Introduction

The progress towards Universal Health Coverage (UHC) involves setting ambitious goals for expanding access to quality health services based on establishing a greater reliance on risk-pooling and prepayment mechanisms to finance health, stimulating investments in healthcare infrastructure and quality, and building human resources and skills for health. The World Health Organization (WHO) estimates that more than half the world´s population does not have access to the health services they need, and 100 million people suffer financial catastrophe every year due to out-of-pocket (OOP) expenditures for unexpected healthcare [1]. Introduction of a health insurance programme is one of the ways to enhance access to healthcare services and to protect individuals from catastrophic health expenditures [2]. Financing healthcare through a tax-based system (which is also a form of risk-pooling) is difficult as many low- and middle-income countries (LMICs) are struggling to mobilize sufficient resources. As a result, OOP expenditures remain high and in combination with poor healthcare services form an important barrier to UHC. How to successfully roll out sustainable health insurance on a large scale and ensure sufficient take-up in LMICs is an outstanding question [3,4].

**Figure 1:**
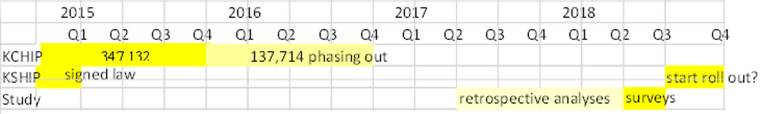
major policy milestone, study period and clients´ enrolment trend in Kwara community health insurance programme between 2015 and 2018

In Africa, more than half of all healthcare expenses are covered through OOP payments. For example, in Nigeria - the most populous country in Africa, with a population of more than 200 million, there are substantial inequalities in access to healthcare with 72% of health expenses paid OOP and only about 4% of the people, mostly in the formal sector, having access to health insurance today [5]. Nigeria accounts for 2% of the world population but contributes to 14% of maternal deaths and 23% of malaria cases [2]. To address these burdens, Kwara State, one of the poorest states in Nigeria, with the support of PharmAccess and the Netherlands Health Insurance Fund launched a subsidized Kwara Community Health Insurance Programme (KCHIP) in 2007 [6-8]. By the year 2015, a total of 347,132 people and 42 public and private healthcare facilities participated in KCHIP (Figure 1).

The impact of KCHIP has been assessed over time through various studies [6-8], indicating an increase in the use of healthcare (while controlling for additional variables) of up to 90% among enrolled communities [7,9], markedly improved cost-effectiveness, as well as substantial benefits in terms of improved health outcomes concerning chronic diseases like hypertension; [10], and maternal and child care [11]. Similarly, OOP expenditures significantly decreased by 50% among enrollees, thus securing more financial protection in the medium run [7,9]. The KCHIP was also found to increase awareness about health status among community members [9,12]. Additionally, it was demonstrated KCHIP could deliver a basic quality healthcare coverage at the US $28 per person per year, compared to the WHO benchmark of US $60 and Nigeria´s total health expenditure per capita of US $115 [8].

An important feature of KCHIP was as incremental financial commitment and ownership of the programme by the Kwara State Government over time. The programme was aimed at synergizing with the Nigeria National Health Insurance Programme (NHIS) to attain UHC for the state [13]. In January 2015 (Figure 1), the programme partners signed an agreement to transition KCHIP to the Kwara State Health Insurance Programme (KSHIP). Pending this arrangement, KCHIP enrolment was temporarily suspended while designing a new insurance product and premium to be introduced and deployed on a state-wide level. Whereas in January 2016, KCHIP was active in 11 out of the 16 Local Government Areas (LGAs) in Kwara State and recorded a total enrolment of 139,714 clients, these clients were not renewed throughout 2016. This resulted in a gradual drop-out over the year with no clients insured by January 2017 (Figure 1). Therefore, a unique ‘reverse insurance intervention´ situation emerged, which was evaluated in this study. This paper describes the consequences of the suspension of KCHIP in Kwara State, Nigeria, in the wake of state-wide health insurance, and analyses the effects on healthcare quality, utilization and financial constraints for healthcare among former enrollees, as well as the consequences for formerly participating KCHIP health facilities.

## Methods

**Study design and study population:** in August 2018, about 2 years after the suspension of KCHIP (Figure 1), a mixed-method study was carried out among KCHIP former enrollees and healthcare providers in Kwara State, Nigeria. Using multi-stage random sampling, we recruited a total of 600 enrollees whose health insurance policy had expired at least 4 months before the end of December 2016. For the quantitative cross-sectional survey we obtain data on socio-demographics, healthcare utilization, enrolment status, health financial constraints and coping strategies since the suspension. Only adults (18 years and above) were included in the study, of whom a purposively selected 400 enrollees had accessed care in a KCHIP healthcare facility in the preceding 12 months. The remaining 200 participants were selected from those uninsured in the past 12 months. Of those 400 participants who have accessed healthcare, half (200) who had in addition to other health conditions been seeking chronic care, maternal care and care for acute conditions were included in the study.

In-depth interviews (IDIs) were performed among 24 purposively selected former enrollees and among 29 health facilities´ managers of (19 public, 10 private) participating KCHIP facilities. The IDIs explored the effects of the programme suspension on both healthcare utilization by former enrollees and their coping mechanisms, and health facilities´ service provision. To be selected for the IDI, the participant must be above 18 years of age and must have utilized pertinent healthcare in the past 12 months. To obtain healthcare utilization pattern due to the programme suspension, health facilities´ clinical records were reviewed as part of the observation checklist tool developed for the qualitative data collection.

### Sampling and data collection

**Quantitative study:** multi-stage sampling was used, selecting 5 Local Government Areas (LGAs): two from Kwara South, two from Kwara North and one from Kwara Central senatorial zones. Enrollees were selected randomly with the KCHIP enrollment database serving as sampling frame after allocating LGAs proportionate to constituent population sizes (total enrollment in the 5 LGAs in January 2016 was 73,438). An additional 30% was added from the sample frame for each LGA to cater for non-response and untraceable enrollees. The selected enrollees were traced in the community (with the help of community mobilizers) and interviewed by trained interviewers. The questionnaire captured data on respondents´ socio-economic characteristics, morbidity patterns, healthcare access and utilization in the preceding 12 months.

**Qualitative study:** we conducted two rounds of IDIs among former enrollees and facilities´ managers. The enrollees´ interviews were conducted among 24 purposively selected adults across 9 selected LGAs cutting across the 3 zones of Kwara State. The selection of former enrollees into the IDIs was carried out in and around the health facilities using a pretested interview guide. The facility managers´ interviews were conducted in KCHIP facilities among the officers-in-charge (or the medical director). This comprised all 29 Enhanced Community Based Care (ECBC) health facilities (19 public, 10 private) spread across 9 LGAs; 13 health posts providing remote care services were excluded from the study because they were already linked to records of the 29 ECBCs.

**Data analysis:**the quantitative data entry platform was designed using Open Data Kit® (ODK), while the data was entered using Kobo Toolbox® [14] and later exported to Statistical Package for Social Science (SPSS) version 22 for analysis. Simple logistic regression was used to explore the predictive factors of the financial constraints in the ability to pay for healthcare services after the programme suspension. The level of significance was set at a p-value of < 0.05 complemented with a 95% confidence interval (CI). Recorded qualitative interviews were transcribed and thematic analysis was carried out manually. Mixed results of the qualitative and quantitative data were triangulated and reported together to complement major contextual observations in this study.

**Ethics approval and consent to participate:** written permissions were obtained from the ethics committee of the Kwara State Ministry of Health, Ilorin, Nigeria. Informed consent was obtained from the participants. Confidentiality of the participants´ and health facilities´ information were maintained.

## Results

**Socio-demography of the enrollees:** the enrollees had a median age of 43 years and 74.5% were women (Table 1). Close to half of the enrollees did not have formal education (42.5%); 77.2% were married and 17.2% widows. About three-quarters of the enrollees (73.8%) lived in semi-urban areas (Table 1). The majority were from Yoruba (64.5%) and Nupe (32.2%) ethnic groups. Islam was the predominant (83.2%) religion amongst them. The enrollees were equally spread over the wealth quintiles, with wealth calculated as annual per capita consumption of food and non-food items.

**Table 1:** socio-demographic and socio-economic characteristics of the respondents

Socio-demography	n (%)	Median (IQR)
Gender		
Male	153 (25.5)	
Female	447 (74.5)	
Age group (years) ≤20	21 (3.5)	
21 - 30	141 (23.5)	
31 - 40	125 (20.8)	
41 - 50	91 (15.2)	
51 - 60	84 (14.0)	
61 - 70	76 (12.7)	
≥ 71	62 (10.3)	
**Median age (years)**		**43 (30)**
**Highest level of education**		
No formal education	255 (42.5)	
Less than primary education	20 (3.3)	
Primary education	77 (12.8)	
JSS education	22 (3.7)	
SSS education	79 (13.2)	
Post-secondary	121 (20.2)	
Quranic education	26 (4.3)	
**Marital status**		
Married	463 (77.2)	
Single	33 (5.5)	
Divorced	1 (0.1)	
Widowed	103 (17.2)	
**Residence**		
Semi-urban	443 (73.8)	
Rural	157 (26.2)	
**Ethnicity**		
Yoruba	387 (64.5)	
Nupe	193 (32.2)	
Hausa	16 (2.6)	
Others	4 (0.7)	
**Religion**		
Islam	499 (83.2)	
Christianity	101 (16.8)	
**Wealth quintile**		
1 (poorest)	126 (21.0)	
2	119 (19.8)	
3	131 (21.8)	
4	108 (18.0)	
5 (richest)	116 (19.4)	

JSS: junior secondary school; SSS: senior secondary school

**Consequences of (re)enrolment suspension on households:** the survey shows that the majority of former enrollees (95.3%) kept utilizing KCHIP facilities, even after the suspension of the programme (Table 2). The factors responsible for this were explored by the IDI. Some former enrollees perceived that the KCHIP facilities have very friendly staff and this would encourage them to keep patronizing the health facilities. However, 74.0% of enrollees reported reverting to OOP payment for healthcare services at the KCHIP facilities. In general, the enrollees had more confidence in the private than the public KCHIP facilities. This is due to full (4.2%) or partial (21.8%) exemption from hospital bill when having financial constraints. According to IDI, many patients had established some friendship and cordial relationships with the facilities throughout the programs. For some enrollees the KCHIP facilities allowed them to pay in tranches for reasons of empathy and familiarity.

**Table 2:** preferences and constraints in ability to pay for and accessibility to healthcare after KCHIP suspension

Preferences/constraints/Coping mechanism	n (%)
**Prefer KCHIP facility**	
Yes	572 (95.3)
No	28 (4.7)
**Current payment options for health services at KCHIP facilities**	
Pay for everything	444 (74.0)
Pay some, but at reduced costs	131 (21.8)
Not pay for anything	25 (4.2)
**Constrained ability to pay for healthcare services**	
No	198 (33.0)
Yes	402 (67.0)
**Main reasons for the constraints (n=402)**	
Due to the suspension	184 (30.8)
Due to the economic situation of the country	68 (11.2)
Due to both the suspension and the economic situation of the country	150 (25.0)
**Coping mechanism (n=600)^**	
From personal savings	380 (63.3)
Donation from friends and families	208 (34.7)
Borrowing (friends/families)	71 (11.8)
Borrowing from local money lenders	4 (0.7)
Sell properties	3 (0.5)
Loan (co-operatives/banks)	4 (0.7)
Support from trade unions	5 (0.8)
Support from religious groups (church or mosque)	4 (0.7)
Have not been ill since program stopped	29 (4.8)
Others identified coping	66 (11.0)
**Other identified coping (n=66)**	
Petty trading	17 (25.8)
Husband	13 (19.7)
Children	3 (4.5)
Others	33 (50.0)
**Accessibility to healthcare since suspension of KCHIP**	
Much worse	78 (13.0)
Worse	296 (49.3)
Same	172 (28.7)
Better	38 (6.3)
Much better	10 (1.7)
Don't know	6 (1.0)
**Membership of financial/social groups (n=600)^**	
Contribution (Ajo)	284 (47.3)
Religious group	171 (28.5)
Community group	112 (18.7)
Cooperative	105 (17.5)
Trade union	32 (5.3)
Social club	49 (8.2)
None of the above	152 (25.3)

^ Multiple responses

We also reported that after suspension, 67.0% of enrollees experienced financial constraints in the ability to pay for healthcare services; 30.8% of whom reported it was due to the suspension, 11.2% to the economic situation of the country while 25.0% said it was due to both (Table 2). But a third (33.0%) of enrollees reported little or no change in their ability to pay for healthcare services. The IDIs reported almost all enrollees had financial constraints their ability to pay for healthcare services. An enrollee said; *“my ability to pay has been hampered seriously by lack of funds”*.

Most common coping mechanisms reported by the enrollees were personal savings (63.3%), donations from friends and families (34.7%) and borrowing (11.8%). Other coping mechanisms included proceeds trading and sales (25.8%), household purse (19.7%) and money from other relatives (4.5%). Most of the enrollees in the qualitative interview agreed that spending household/family savings to offset healthcare bills were the immediate coping mechanism available to them after the programme suspension. Others narrated that they had to borrow from friends and family members, including seeking assistance from other relatives including the children in paying hospital bills. Some prominent social group mechanisms that offered health benefits to members such as donations during episodes of illness and loan facilities to offset medical bills were reported from the quantitative data. Examples of these mechanisms were (Table 2): Ajo - a local thrift (47.3%), religious groups (28.5%), community groups (18.7%) and cooperative groups (17.5%).

**Factors associated with a constrained ability to pay for healthcare among enrollees after the programme suspension:** different factors were found to be associated with the reported financial constraints inability to pay for healthcare after insurance suspension (Table 3). Constraints were experienced more often by male enrollees (74.5%, p = 0.022), living in rural locations (75.8%, p = 0.006) and by those having an acute illness/injury in the preceding 12 months (74.3%, p<0.001). Besides, ethnic groups other than Yoruba (Nupe 89.6% and Hausa 87.5%) in the study (p<0.001) and those enrollees that patronized KCHIP facilities exclusively before suspension (68.1%, p<0.001) were significantly more likely to be financially constrained to pay for healthcare services after the programme suspension. Wealth was also significantly associated with constraint ability to pay. Being in lower wealth quintiles is associated with constraints ability to pay for healthcare.

**Table 3:** factors associated with constraints in ability to pay for healthcare services after re-enrollment suspension

Factors	Constrained ability to pay		X^2^	p-value
	Yes n (%)	No n (%)		
**Gender**			**5.238**	0.022*
Male	114 (74.5)	39 (25.5)		
Female	288 (64.4)	159 (35.6)		
**Residence**			**7.441**	0.006*
Semi-urban	283 (63.9)	160 (36.1)		
Rural	119 (75.8)	38 (24.2)		
**Ethnic group**			**74.025**	< 0.001*
Yoruba	212 (54.8)	175 (45.2)		
Nupe	173 (89.6)	20 (10.4)		
Hausa	14 (87.5)	2 (12.5)		
Others	3 (75.0)	1 (25.0)		
**Wealth quintile**			**16.739**	0.002*
1	98 (77.8)	28 (22.2)		
2	87 (73.1)	32 (26.9)		
3	86 (65.6)	45 (34.4)		
4	66 (61.1)	42 (38.9)		
5	65 (56.0)	51 (44.0)		
**Perceived reasons for the KSCHIP suspension**			**19.949**	0.001*
Government failure to provide funds	142 (62.6)	85 (37.4)		
Hygeia no longer fund the program	99 (83.9)	19 (16.1)		
Stoppage of foreign fund	3 (42.9)	4 (57.1)		
No reason given	139 (67.5)	67 (32.5)		
Others	9 (56.2)	7 (43.8)		
**Utilization of non-KSCHIP facility while in the program**			**4.365**	0.037*
Yes	22 (52.4)	20 (47.6)		
No	380 (68.1)	178 (31.9)		
**Suffering for any acute illness or injury in last 12 months**			**41.434**	<0.001*
Yes	33 (74.3)	114 (25.7)		
No	72 (46.2)	84 (53.8)		

* Statistical significance; X^2^: chi-square

Per Table 4, the predictive factors for being financial constrained were being male (OR=1.61, 95% CI=1.069; 2.436) and living in rural communities (OR=1.77, 95% CI=1.171; 2.677). Enrollees of Yoruba ethnicity (OR=0.15, 95% CI=0.091; 0.236) had less financial constraints in paying for healthcare services after the programme suspension compared to people from other ethnicities. Those enrollees who depended solely on KCHIP health facilities before suspension had increased odds (OR=1.94, 95% CI=1.032; 3.648) of financial constraints while those with acute illness or injury in the preceding 12 months also had increased odds (OR=3.38, 95% CI=2.309; 4.939).

**Table 4:** predictors of constrained ability to pay for healthcare services after the suspension of the program

Factor	β	p-value	Crude OR	95% lower	CI upper
Male gender	0.479	0.023*	1.614	1.069	2.436
Age	0.004	0.454	1.004	0.994	1.013
Rural inhabitants	0.571	0.007*	1.771	1.171	2.677
Yoruba ethnic group	-1.920	<0.001*	0.147	0.091	0.236
Higher wealth quintiles	-0.255	<0.001*	0.775	0.685	0.877
Utilize only KSCHIP facilities while in the program	0.663	0.039*	1.941	1.032	3.648
Enrollees with acute illness or injury	1.217	<0.001*	3.377	2.309	4.939
Enrollees with chronic illness	0.260	0.154	1.298	0.907	1.856
Membership of financial group	0.379	0.068	1.461	0.972	2.195

* Significance level (p) < 0.05

**Figure 2:**
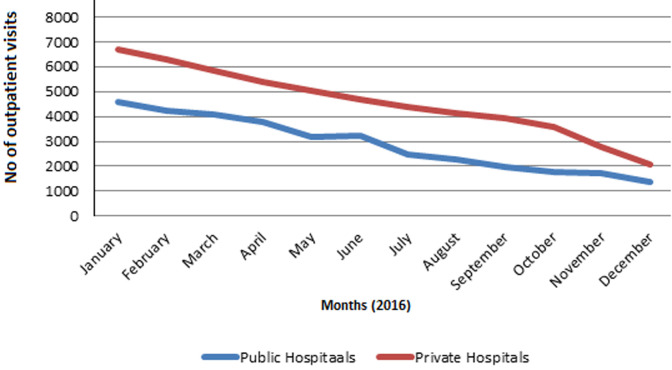
out-patients´ visits by month for the year 2016 across the public and private health facilities

**Figure 3:**
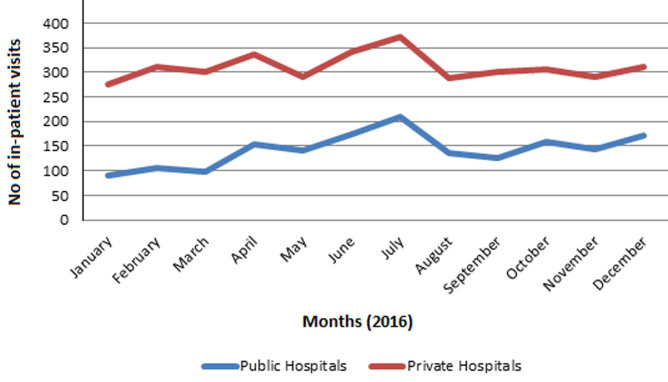
in-patient visits by month for the year 2016 across public and private health facilities

**Consequences of the programme suspension on the KCHIP facilities (IDI and hospital records):** after the suspension, 24 of 29 health facilities claimed the quality and quantity of services provided remained the same while five confirmed reduction in service provision. In the past, more than two-thirds of the health facilities claimed they experienced increased patronage and service utilization due to KCHIP. However, with the suspension of the program, records revealed that all facilities experienced a significant reduction in out-patient loads as there was a gradual decline in healthcare utilization (Figure 2). No appreciable effect was seen on in-patient visits (Figure 3). Out of those that reported a reduction in service provision, a facility manager commented that: *“at present, just about 5% of those previously registered on the programme is still coming to the health facility for treatment”*. Another manager narrated that: *“We saw just 2 patients today, compared to the time when the programme was in place whereby we will not have the time to even attend to you to have this interview”*.

Seventeen of 29 facilities reported a decrease in revenue after suspension of the program. Nine recorded an increase in their revenue, despite a reduction in patient load; the facilities in this category were all public health facilities. They attributed this increase to the removal of restrictions on billing patients directly during the enrollment period. A facility manager said: *“Our revenue has increased because patients now have to pay out of pocket unlike when the programme was running and drugs and tests were done freely”*. Another said: *“Our revenue has increased because people coming to the hospital now pay for services”*. Similarly, 23 out of 29 facilities experienced a high staff turnover after suspension of KCHIP. Almost 90% (26) of the facilities reported a salary cut for their staff after the suspension. One of the facility managers recounted that: *“with the suspension of the program, the revenue generated could not cater for the salaries of all the staff so 39 members of staff were laid off and those that remained (31) had a 40% salary cut”*. However, the loss of incentives and salary cut reduced staff motivation and productivity as attested to by a manager: *“the salary cut, as well as the loss of incentives, has resulted in low productivity of staff members who claim not to be motivated anymore”*. More facilities (21) informed of the decrease in drug purchase after suspension of the program.

## Discussion

This study assessed the effects of the suspension of a community-based health insurance programme in Kwara State, Nigeria. This suspension was due to the restructuring and repositioning of the scheme for a major government policy change to herald a state-wide health insurance program. This, therefore, necessitated an unusual 'reverse intervention' evaluation of community-based health insurance. This we carried out using a mixed-method to study both the former enrollees in KCHIP and the participating healthcare facilities.

Firstly, this study reported that despite the suspension of KCHIP, the large majority of former enrollees still preferred to use the KCHIP health facilities. This was mostly due to extended positive experiences and relationships previously established with the KCHIP health facilities and perceived quality of care. The quality upgrades and periodic training drive at the KCHIP facilities before the programme suspension were most likely contributory factors to the quality of care observed by the enrollees. Also, other quality improvement interventions executed by KCHIP included capacity building on protocol and guidelines for treatments, records, laboratory, drug storage and infrastructure [10,11]. Therefore, this improved the quality of care and standard of practice in the KCHIP facilities with basically a few alternative options of similar medical quality available for enrollees in the state. The trust and the continuing usage of the KCHIP facilities reported by the enrollees after the suspension showed the propensity of community-based health insurance, when combined with quality improvement of medical services, to remove barriers to healthcare utilization [4]. The trust and cordial relationship are shown by the former enrollees to KCHIP facilities can be harnessed for the high uptake of the incoming state-wide health insurance scheme.

This study also demonstrated that three-quarters of the former KCHIP enrollees reverted to OOP payment after suspension of the program. Because of the established relationships with KCHIP facilities, the remaining one-quarter of enrollees were treated for free or were allowed to make partial or tranche-wise payments, even with private healthcare providers. This could be an indicator of the build-up of social benefits from the KCHIP, though at a certain cost to the healthcare providers. The high rate of reversal to OOP endangered and eroded the original insurance aspirations and benefits of KCHIP [6,8,10] and it also represents a potential threat, which can plunge enrollees into catastrophic health expenditure [8,15].

We show that of the two-thirds of former enrollees who experienced constraints to pay for healthcare services, the suspension, as well as the general economic recession in Nigeria, were mentioned as the most important perceived causes. The economic recession has been reported elsewhere to cause a reduction in individual expenditure and health insurance consumption [16]. We found that the KCHIP suspension had additional and immediate consequences for former enrollees, leading to the adoption of financial coping mechanisms like personal savings, donations and borrowing. Enrollees also reported receiving support from financial or social groups in the form of “Ajo” thrifts and; religious, community and social cooperative groups as a form of coping for the unexpected healthcare cost after the suspension. These were beneficial to individuals who required funds for sickness. Such financial and social groups are effective coping strategies in terms of improved household income [17]. This underscores the potentials of local thrifts and cooperative groups in financing the health insurance enrollment fees in Nigeria communities.

The male enrollees living in rural communities reported more difficulties paying for healthcare services after the programme suspension. This is in line with a study on catastrophic health expenditure in Nigeria, which concluded that female-headed households were less likely to incur catastrophic expenses compared to male-headed households [18]. This reflects lower access to healthcare services and higher foregone formal care among women compared to men [19,20]. The Yoruba ethnic group appeared less constrained to pay for healthcare services after the suspension. Living in rural communities of Nigeria is associated with poverty, poor infrastructure and lack of geographical and financial access to healthcare services [21]. Our findings on the wealth quintiles that indicated a significant socio-economic gradient in access to healthcare after suspension looks similar to the inference by another local study [22], which concluded that the richer quintiles indeed experienced less catastrophic health expenditure.

A previous study in Kwara State on spending for non-communicable chronic disease (NCCD) reported health expenditures relative to the annual consumption of the poorest quintile exceeding those of the highest quintile 2.2-fold, and the poorest quintile exhibiting a higher rate of catastrophic health spending (10.8% among NCCD-affected households) than the three upper quintiles (4.2% to 6.7%) [19]. This finding to the state-wide scheme implies that the low socio-economic group are at more risk of financial constraint. They should have enrollment fees subsidized or paid for through a government social scheme. Enrollees who experienced an acute illness or injury in the preceding 12 months before the suspension of enrollment had increase odds of being financially constrained in the ability to pay after the suspension. Several Nigerian studies [22] also reported an increased risk of incurring catastrophic health expenditures for household members with non-chronic illnesses. This finding implied that the enrollees with acute illnesses are more likely to be unprepared and could suffer more financial constraints paying for healthcare services without health insurance scheme.

While there were no serious consequences concerning the range of service provision, a significant reduction in patient load in (almost) all of the KCHIP facilities were observed. However, there were slight spikes on patient load around the wet months of the year, which supported seasonal patterns of health-seeking behavior in Nigeria. This is mostly related to malaria season and harvest time. All health facilities´ revenues dropped considerably as enrollees exited the program. Private health facilities experienced higher drops in revenue after KCHIP suspension. Public facilities still received stipends from the government to run their services, which cushioned these effects. Some public health facilities even reported an increase in the revenue generation because of the removal of insurance programme users charge restrictions on the direct billing of the patients. This is also possibly due to some shift of private patients towards the public sector since some services were free in public health facilities. Private health facilities disproportionately suffered a reduction in staff strength, motivation and productivity. This resulted in the downsizing of staff in many of these facilities. Also, we observed a downward trend in drug purchase among the private health facilities, which remained unchanged in public facilities that kept benefiting from the supply of essential drugs from the ministry of health. Finally, the suspension of KCHIP was reflected by clear downward out-patient department visits, but in-patient visits remained the same. These finding revealed that though the private facilities enjoyed the trust of the enrollees before the suspension, they were unable to cope with service provisions and staff retention after suspension like the public facilities who enjoyed funding from the government.

In the literature, suspending an impactful health insurance programme is an unusual policy decision. This is probably due to high political sensitivity and the legislative bureaucracy that such action will cause. In January 2016, the Qatari government suspended a state-financed mandatory national health insurance programme due to inability to sustain the exclusive funding of the programme because of a fall in global oil prices [23]. Experts expected in the short term a larger private sector involvement in the Qatari healthcare coverage, while in the long term and uncertainty regarding payment of Qataris' medical bills and UHC. The suspension of the Qatari health insurance programme adversely affected hospitals, health centers and patients, which caused a negative outcry among the population [23]. Similar observations are made in Kwara concerning deteriorating access to healthcare, which happened much rapidly due to the weaker healthcare infrastructure and poverty status of the population. Shifting from fragmented smaller-scale community-based health insurance programs to a larger state-owned insurance programme is a precarious process. Lessons can be learnt from elsewhere in Africa, like the development of the National Health Insurance Scheme (NHIS) in Ghana [24], the political path to impactful community health insurance in Rwanda [25] and the transition of the improved Community Health Fund (iCHF) into a national iCHF in Tanzania [26]. A common recommendation is the introduction of a transition phase with clearly defined services before the new larger-scale insurance package is introduced, providers are assigned and financial coverage is arranged for instance through tax systems, like value added tax (VAT) such as in Ghana [24].

This paper demonstrates that temporary suspension of health insurance in the absence of transitional measures has consequences for enrollees and healthcare providers. It also provides opportunities to learn lessons. For example, it was learnt that transition periods can leverage on previously built social capital, including the network of relations between former enrollees and healthcare providers, as well as the support from particular social groups (religious, community and cooperatives). It was also learnt that refurbishment of health facilities and quality improvement of services during the previous phase of community-based health insurance was appreciated also during the suspension of the KCHIP, with people continuing to visit KCHIP healthcare facilities. At the policy level, Kwara State worked to adopt a law that makes health insurance mandatory for all inhabitants and requires that the state government commits one percent of its revenues to finance health insurance. In addition, during the transition phase, Kwara State started the process of setting up a dedicated state health insurance fund that pools financial contributions from diverse sources, including the State Government, the Federal Government of Nigeria (particularly Ministry of Health and National Health Insurance Program) and individual enrollees. All these should be harnessed for a sustainable and effective health insurance scheme in Kwara State, Nigeria.

## Conclusion

After the suspension of the KCHIP health insurance programme in Kwara State, Nigeria, former enrollees still preferred using the KCHIP health facilities and they reverted almost ubiquitously to OOP payments. At the same time, out-patient healthcare consumption decreased substantially, with a large proportion of former enrollees not being able to afford healthcare services. Belonging to some form of financial/social group proved beneficial in the short term as a coping mechanism. Social capital built through KCHIP between former enrollees and clinics helped alleviate part of the financial burden for the former enrollees, but not for the facilities. Enrollees with the highest probability of suffering adverse consequences of the programme suspension were male enrollees, households in the lower social quintiles, living in rural communities and those reporting recent acute illness. Private health facilities suffered more consequences of the programme suspension than public facilities in terms of reduced financial inflow sequel to change in the revenue and resources. These observations point to the need for designing effective transition processes from community-based health insurance to state insurance in Nigerian states.

**Funding:** though the PharmAccess Foundation funded this study and one of the authors work at the company, the study was not influenced by his participation in the design, data collection and manuscript writing.

### What is known about this topic

- It is already known fact that community-based health insurance scheme is an important strategy to achieve accessibility to quality healthcare for underserved population; Kwara Community Health Insurance Scheme was adjudged as one of the impactful health intervention from sub-Saharan Africa.

### What this study adds

- This study provides insight into the consequences of suspending an impactful health intervention like Kwara Community Health Insurance Scheme.
- It also highlighted the common challenges experienced and coping strategies adopted by the enrollees and the care providers on the scheme.
